# New *Atanatolica* species from Ecuador (Trichoptera, Leptoceridae)

**DOI:** 10.3897/zookeys.793.26712

**Published:** 2018-10-29

**Authors:** Ernesto Rázuri-Gonzales, Ralph W Holzenthal, Blanca Ríos-Touma

**Affiliations:** 1 Department of Entomology, University of Minnesota, 1980 Folwell Avenue, 219 Hodson Hall, St. Paul, Minnesota 55108, USA University of Minnesota St. Paul United States of America; 2 Facultad de Ingenierías y Ciencias Aplicadas, Ingeniería Ambiental, Grupo de Investigación en Biodiversidad Medio Ambiente y Salud – BIOMAS – Universidad de Las Américas, Campus Queri, Quito, Ecuador Universidad Nacional Mayor de San Marcos Lima Peru; 3 Departamento de Entomología, Museo de Historia Natural, Universidad Nacional Mayor de San Marcos, Lima, Perú Universidad de Las Américas Quito Ecuador; 4 Instituto Nacional de Biodiversidad, Quito, Ecuador Instituto Nacional de Biodiversidad Quito Ecuador

**Keywords:** Andes, aquatic insects, Grumichellinae, long-horned caddisflies, taxonomy

## Abstract

Four new species of *Atanatolica* Mosely are described from Ecuador: *A.andina***sp. n.**, *A.angulata***sp. n.**, *A.curvata***sp. n.**, and *A.decouxi***sp. n.** These species belong to the *A.dominicana* group and constitute new records of the genus from Chimborazo, Imbabura, and Napo Provinces. Additionally, *A.andina***sp. n.** represents the highest elevation recorded for any species in the genus at 3900 m. Size class data are also presented suggesting continuous larval growth for the probable larva of *A.decouxi***sp. n.**, described and illustrated here. A new distribution record is provided for *A.manabi* from Carchi Province.

## Introduction

*Atanatolica* Mosely, 1936 is a Neotropical genus in the long-horned caddisfly family Leptoceridae. Originally, the genus was established to include a single species, *Mystacidesbrasilianus* (Brauer, 1865), based on characters of the wing venation and male genitalia (Mosely 1936). Only *A.dominicana* Flint, 1968 and *A.botosaneanui* Flint, 1981 were described before Holzenthal’s 1988 revision, which included descriptions of 14 new species and redescriptions of the previously described ones. More recently, four new species were described from Brazil and Peru (Costa and Calor 2014, Henriques-Oliveira and Santos 2014, Oláh 2016), bringing the current number of species in the genus to 21 (Holzenthal and Calor 2017). Holzenthal (1988) recognized two species groups, the *A.brasiliana* and the *A.dominicana* groups. The *A.brasiliana* group has a sessile fork I in the forewing while this fork is petiolate in *A.dominicana* group species.

Fourteen species of *Atanatolica* are known from the northern and central Andean countries (Venezuela, Colombia, Ecuador, Peru, and Bolivia); no species are known from the southern Andes (Chile, Argentina) (Holzenthal and Calor 2017, Oláh 2016). Four species are known from Brazil (two in the southeast, two in the northeast), two from Central America, and one from the Lesser Antilles. None of these species, except *A.dominicana*, are present in more than one country, and most are described from very few adults (Holzenthal 1988). Currently, there are three species known from Ecuador (Ríos-Touma et al. 2017): *A.acuminata* Holzenthal, 1988, *A.cotopaxi* Holzenthal, 1988, and *A.manabi* Holzenthal, 1988, all of them in the *dominicana* group.

Larvae are associated with small and medium-sized Neotropical mountain streams, waterfalls, their splash zones, and even outside the water in moist, semiterrestrial habitats (Flint 1968, Holzenthal 1988). Larvae and pupae are usually found in large numbers, often in groups, attached to the substrate by anterior silken pedicles or with silken strands attached to rocks to improve larval purchase and mobility on smooth surfaces (Holzenthal 1988). Ecologically, they are considered scrapers, probably feeding on periphyton and deposited organic matter (Holzenthal 1988, Holzenthal and Calor 2017). Adults are diurnal and form swarms above larval habitats, and therefore, they are not common at UV light traps commonly used to attract caddisflies (Holzenthal and Calor 2017). *Atanatolicabonita* Costa & Calor, 2014 is the only species in the genus for which there is any seasonal information available. Abundance of this species fluctuated through the year, with increasing abundance in the dry season, at least in the Brazilian region surveyed by Costa and Calor (2014).

Here we describe four new species of *Atanatolica* from Ecuador. These new species come from several localities along the Andes: *A.andina* sp. n. from the highlands of the Amazon drainage, *A.decouxi* sp. n. from the cloud forests of the Pacific drainage of the Andes, and *A.angulata* sp. n. and *A.curvata* sp. n. from the Amazon piedmont. For *A.decouxi* sp. n. we also describe the probable larva and provide size class information and some biological observations.

## Material and methods

### Localities and collecting methods

**Figure 1.**
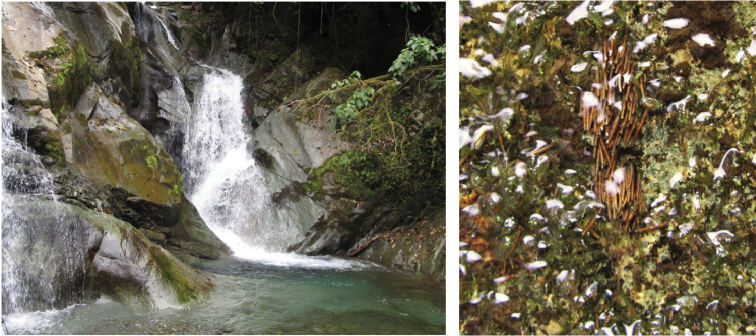
**A** Río de la Plata, Reserva Los Cedros, type locality of *Atanatolicadecouxi* sp. n. **B** Same, larvae of *A.decouxi* sp. n. on stream substrate.

*Atanatolicaandina* sp. n. was collected at high-altitude waterfalls surrounded by *páramo* vegetation in Parque Nacional Cayambe-Coca (Napo Province) and Parque Nacional Sangay (Chimborazo Province) using aerial nets. Additional specimens were collected by B. Gill, also at Parque Nacional Cayambe-Coca. Specimens of *Atanatolicadecouxi* sp. n. were collected at Río de la Plata, a pristine stream in the Bosque Protector los Cedros (Imbabura Province) using UV lights and Malaise traps for adults and Surber nets and hand collecting for immatures. Bosque Protector los Cedros is part of the Choco-Darien floristic region, which is considered a biogeographic hotspot and priority conservation area due to its high species richness and endemism (Dodson and Gentry 1991, Myers et al. 2000). At the collection site the river has a small waterfall followed by two deep clear pools (Figure 1). The two other described species were borrowed from the Smithsonian Institution and were collected by O. S. Flint Jr. in the Amazon piedmont of Napo Province in 1990.

Locality data were formatted using the web application AUTOMATEX (Brown 2013) to increase consistency. The map was prepared in QGIS 3.2.2. Bonn (QGIS Development Team 2018). Vector and raster maps were made with Natural Earth (2018) and CIAT-CSI SRTM (Jarvis et al. 2008) data.

### Specimen preparation

Adult specimens were prepared and examined following standard methods for pinned and alcohol preserved material (Blahnik and Holzenthal 2004, Blahnik et. al. 2007). Length of forewing was measured from base to apex with a microscale (BioQuip Products, Rancho Dominguez, California, USA). Body length, head width and length, and case length of all larvae collected (n = 89) were measured to establish size variation and stages across the collecting dates (as described by Resh 1976). Measurements were performed with the aid of a Zeiss V12 Discovery Stereoscope with an Axiocam ICc5 camera and the Axiovision SE64 software.

Male genitalia were soaked in 85% lactic acid and heated to 125 °C for 20 min to dissolve internal soft tissues. Olympus BX41 and SZX12 compound and stereomicroscopes outfitted with drawing tubes were used to examine specimens and to aid the rendering of detailed pencil drawings of genital structures and larvae, respectively. Pencil sketches were scanned and placed in Adobe Illustrator (Creative Cloud version) to serve as a template for vector illustrations. The plugin “Stipplism” (Astute Graphics) was used to apply stipple effects to illustrations. Morphological terminology follows that of Holzenthal (1988).

Types of the new species and other material examined are deposited in the University of Minnesota Insect Collection, St. Paul, Minnesota, USA (**UMSP**), the Museo Ecuatoriano de Ciencias Naturales, Instituto Nacional de Biodiversidad, Quito, Ecuador (**MECN**), and the National Museum of Natural History, Smithsonian Institution, Washington, DC (**NMNH**). Each specimen housed at UMSP or MECN was affixed with a barcode label (4-mil polyester, 8 × 14 mm, code 49) bearing a unique alphanumeric sequence beginning with the prefix UMSP to serve as a specimen identifier (UID) for upload of collection and specimen data to the UMSP database; UIDs for holotypes deposited in UMSP and NMNH are listed in the material examined.

## Taxonomy

### Atanatolica andina sp. n.

Taxon classificationAnimaliaTrichopteraLeptoceridae

http://zoobank.org/A2D13672-1B8E-4D08-BA72-63A1BF05AE32

Figs 2 , 8

#### Diagnosis.

This new species is most similar to *A.acuminata* and *A.dominicana* from Ecuador and Dominica, respectively, based on the general structure of tergum X (i.e., subtriangular in shape, with digitate apicomesal processes). It differs from *A.acuminata* by the shorter, thicker apicomesal processes on tergum X, and the shorter and rounder apicolateral processes on tergum X; in lateral view, the apicolateral processes in *A.andina* sp. n. are much shorter than the apicomesal processes, whereas in *A.acuminata*, both processes are roughly equal in length and the apicolateral processes are accuminate. Additionally, the inferior appendages in *A.andina* sp. n. are inflated mesally in lateral view, but not in *A.acuminata*. From *A.dominicana*, it differs by having a much narrower and shallower mesal cleft on tergum X between the apicomesal processes. Additionally, the posteromesal margin of the inferior appendages in ventral view is rounder and more pronounced in *A.andina* sp. n., whereas in *A.dominicana*, this margin is straight.

#### Description.

**Adult male.** Forewing length 9.8 ± 0.5 mm (n = 3). General color black, forewing membrane brown, covered in brown and white setae. Head with long, brown setae. Antennae with long, brown setae on scape and pedicel, flagellomeres with dark brown setae and ring of white setae basally. Maxillary palps brown, with long, brown hairs. Thorax black, with dark brown hairs. Forelegs brown, tarsomeres with white ring basally; mid legs dark brown with white setae; hind legs dark brown with white setae, interspersed with brown spines, increasing in thickness towards the tarsal segments. Tibial spur formula 0, 2, 2.

#### Genitalia.

**Figure 2.**
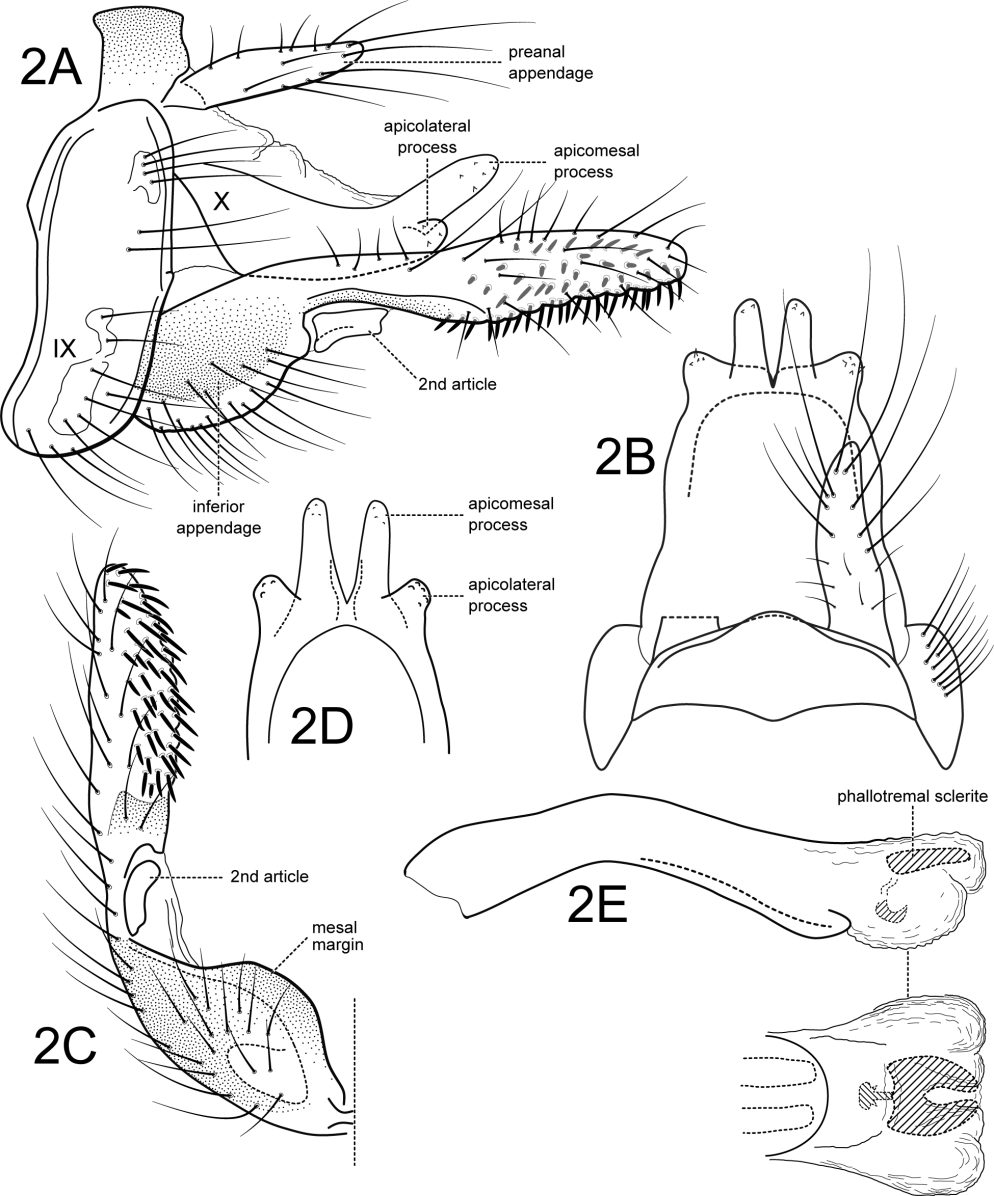
*Atanatolicaandina* sp. n., male genitalia. **A** Lateral **B** Segments IX and X, dorsal **C** Inferior appendage, ventral **D** Tergum X, caudoventral **E** Phallic apparatus, lateral (inset: phallic apparatus apex, ventral). Abbreviations: IX abdominal segment IX, X abdominal tergum X.

Segment IX annular, short, with anterior margin sinuous, posterior margin slightly produced mesally (Figure 2A). Preanal appendages shorter than tergum X, slender, digitate, setose (Figure 2A, B). Tergum X slightly notched apicomesally, basal portion of tergum X membranous, with two pairs of sclerotized apical processes; apicomesal processes digitate and directed upwards in lateral view (Figure 2A); apicolateral processes much shorter than apicomesal processes, thumb-like, directed laterad in dorsal and caudoventral views (Figure 2B, D); apices with short spicules (Figure 2A, B, D). Inferior appendages with basal portion of first segment broad, setose, highly pigmented, mesal margin rounded, apical portion digitate, straight, slightly inflated subapically in ventral view (Figure 2C), covered with stout, spine-like setae on its mesal surface; second article short, rectangular (Fig. 2A, C). Phallic apparatus simple, without any processes; phallobase tubular; phallotremal sclerites complex, consisting of pair of elongated sclerites basally (very faint in the specimens examined), hooked sclerite subapically, and U-shaped sclerite apically (rectangular in lateral view); endothecal membranes trilobed (Figure 2E).

#### Holotype male.

**ECUADOR**: **Napo**: Reserva Ecológica Cayambe-Coca waterfall, rd. to Oyacachi, 0.32621S, 78.1505W, 3690 m, 26.ii.2012, B Ríos-Touma, L Pita (UMSP) [UMSP000098741].

#### Paratypes.

**ECUADOR**: **Chimborazo**: small roadside waterfall on Highway E-46 (via Riobamba - Macas), 2.17572S, 78.5047W, 3527 m, 2♂, 25.i.2015, R Holzenthal, B Ríos-Touma (MECN); **Napo**: unnamed trib. to Oyacachi R., ca. 5.2 mi W of Oyacachi, 0.229504S, 78.0059W, 2823 m, 1♂, 24.ii.2012, B Gill (NMNH), unnamed tributary to Papallacta River, Hwy. E-28, ca. 1 km SW Papallacta, 0.38589S, 78.1435W, 3246 m, 8♂, 6♀, 25.i.2012, B Gill (NMNH), stream, 2.73 Km W Papallacta, Hwy. E-28, 0.534639S, 78.2254W, 3982 m, 8♂, 25.i.2012, B Kondratieff, B Gill (NMNH).

#### Etymology.

Named after the Andean ranges where the specimens were collected.

#### Distribution.

Napo and Chimborazo Provinces (Ecuador) (Figure 8). The species occurs at the highest elevation ever recorded for the genus.

### Atanatolica angulata sp. n.

Taxon classificationAnimaliaTrichopteraLeptoceridae

http://zoobank.org/4FFF866C-42EA-4014-BFFD-091D54B755A1

Figs 3 , 8

#### Diagnosis.

This species is related to *A.aurea* Holzenthal, 1988 from Colombia, and *A.penai* Holzenthal, 1988 from Bolivia, especially in the broad apicomesal processes of tergum X. The new species differs from *A.aurea* by the much shorter, acute, angulate apicomesal processes, and the rounded apicolateral processes on tergum X as well as the shape of the inferior appendages, especially the posteromesal margin of the first segment of this structure in ventral view. From *A.penai*, it differs by the shape of the apicomesal processes of tergum X, which in *A.angulata* sp. n. are strongly angulate. They also differ by the shape of the inferior appendages, which have a mesal bump on the inner surface in *A.penai*, but not in *A.angulata* sp. n.

#### Description.

**Adult male.** Forewing length 9 mm (n = 1). General color light brown, forewing membrane brown, covered in golden and brown setae. Head with long, white setae. Antennae with long, white setae on scape and pedicel, flagellomeres with dark brown setae and ring of white setae basally. Maxillary palps light brown, with long, white and short brown hairs. Thorax light brown with brown hairs. Forelegs with coxae, trochanter, and femur light brown with long, white setae; tibia and tarsomeres with white and brown setae; mid and hind legs light brown with white and brown setae, tibia and tarsomeres with two rows of dark spines ventrally. Tibial spur formula 0, 2, 2.

#### Genitalia.

**Figure 3.**
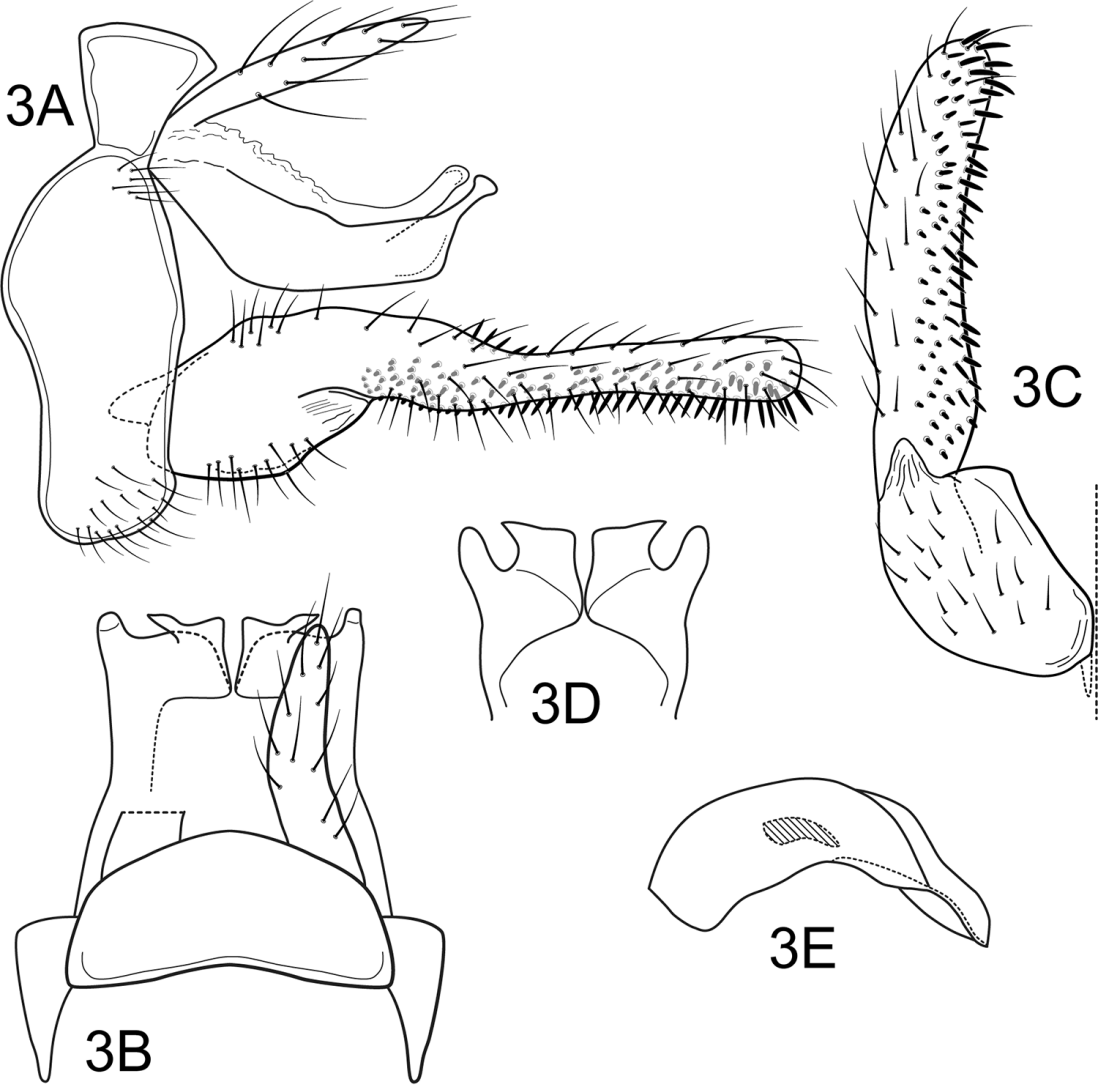
*Atanatolicaangulata* sp. n., male genitalia. **A** Lateral **B** Segments IX and X, dorsal **C** Inferior appendage, ventral **D** Tergum X, caudoventral **E** Phallic apparatus, lateral.

Segment IX annular, short, with anterior and posterior margins sinuous, setae on ventral and lateral surfaces not associated with warts (Figure 3A). Preanal appendages slightly shorter than tergum X, slender, digitate, setose (Figure 3A, 3B). Tergum X notched apicomesally, basal portion of tergum X membranous, with two pairs of sclerotized apical processes; apicomesal processes flattened, directed dorsad in lateral view (Figure 3A), strongly angulate, posterolateral apex acute in dorsal and caudoventral views (Figure 3B,) ; apicolateral processes capitate, directed dorsad in lateral view, slightly longer than the apicomesal processes, but equally as long in dorsal and caudoventral views (Figs 3A, B, D), digitate in dorsal and caudoventral views (Figure 3B, D). Inferior appendages with basal portion of first segment broad, setose, mesal margin forming an angle, apical portion elongate, digitate, slightly curved, and slightly inflated basally in ventral view (Figure 3C), covered with stout, spine-like setae on mesal surface; second article apparently fused with the first segment, represented by triangular semimembranous process (Figure 3A, C). Phallic apparatus simple, without any processes; phallobase short, tubular, curved downwards; phallotremal sclerite simple, spine-like, curved; endothecal membranes not everted (Figure 3E).

#### Holotype male.

**ECUADOR**: **Napo**: Río Jondachi, 30 km N Tena, 950 m, 10.ix.1990, OS Flint (NMNH) [USNMENT01295341].

#### Etymology.

The specific name *angulata* is a Latin adjective referring to the angulate apicomesal processes on tergum X.

#### Distribution.

Napo Province (Ecuador) (Figure 8).

### Atanatolica curvata sp. n.

Taxon classificationAnimaliaTrichopteraLeptoceridae

http://zoobank.org/2291EBFF-73FF-4F10-A776-C426797601AA

Figs 4 , 8

#### Diagnosis.

**Figure 4.**
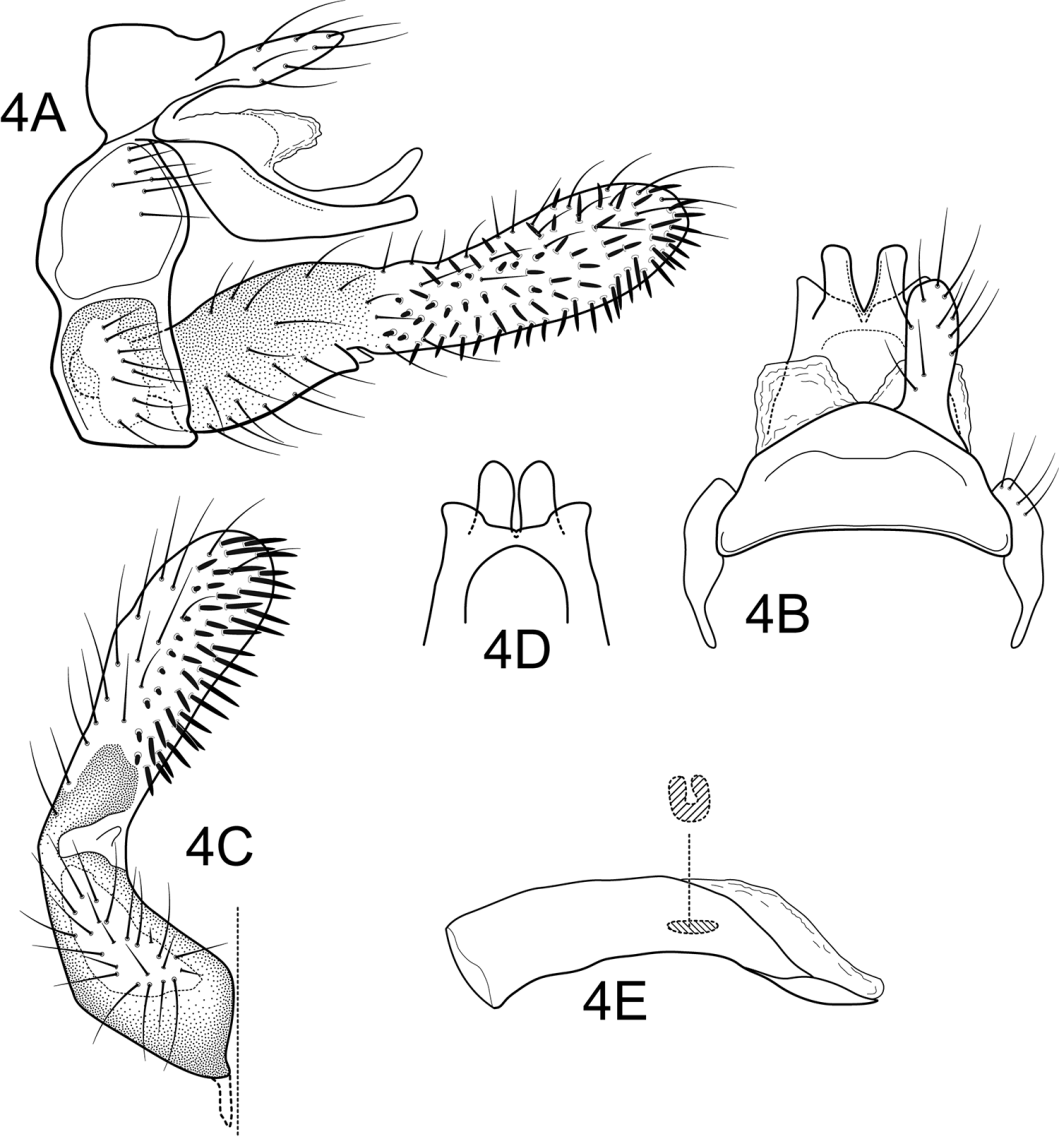
*Atanatolicacurvata* sp. n., male genitalia. **A** Lateral **B** Segments IX and X, dorsal **C** Inferior appendage, ventral **D** Tergum X, caudoventral **E** Phallic apparatus, lateral (inset: phallotremal sclerite, ventral).

This new species resembles *A.homora* Oláh, 2016 from Peru in that both possess paired, basodorsal membranous lobes on tergum X, but in *A.homora* these lobes bear peg-like setae and are narrower than in *A.curvata* sp. n. Also, the apicomesal processes of tergum X are long and capitate in *A.homora* (Oláh 2016, fig 26), but short and digitate in *A.curvata* sp. n. (Figure 4B, D).

#### Description.

**Adult male.** Forewing length 10.5 mm (n = 1). General color light brown, forewing membrane light brown, covered in golden setae throughout wing membrane and brown setae on costal margin. Head with long, yellow setae dorsally, brown setae ventrally. Antennae broken at second flagellomere, scape and pedicel with long, light brown setae. Maxillary palps light brown, with long, brown hairs. Thorax light brown with long, yellow setae. Forelegs light brown, tibia with brown setae, tarsomeres with white ring basally; mid and hind legs with yellow setae, and two rows of dark spines on tibia and tarsomeres. Tibial spur formula 0, 2, 2.

#### Genitalia.

Segment IX annular, short, with anterior and posterior margins sinuous, with setae on ventral and lateral surfaces (ventral setae arising from a wart) (Fig. 4A). Preanal appendages shorter than tergum X, slender, digitate, setose (Figure 4A, 4B). Tergum X slightly notched apicomesally, basodorsal portion of tergum X membranous, produced into pair of membranous lobes; with two pairs of sclerotized apical processes; apicomesal processes digitate and directed dorsad in lateral view (Figure 4A); apicolateral processes shorter than apicomesal processes, apex subtriangular in dorsal view, directed laterad in dorsal and caudoventral views (Figure 4B, 4D). Inferior appendages with basal portion of first segment broad, setose, highly pigmented, mesal margin concave in ventral view (Figure 4C), apical portion digitate, curved mesad, inflated apically in ventral view, covered with stout, spine-like setae on its mesal surface; second article very small, short, triangular (Figures 4A, 4C). Phallic apparatus simple, without any processes; phallobase tubular; phallotremal sclerite simple, U-shaped in ventral view; endothecal membranes not everted (Figure 4D).

#### Holotype male.

**ECUADOR**: **Napo**: 12 km W Baeza, 2380 m, 09.ix.1990, OS Flint (NMNH) [USNMENT01295342].

#### Etymology.

The specific name *curvata* is a Latin adjective that means curved and refers to the strongly curved inferior appendages in ventral view.

#### Distribution.

Napo Province (Ecuador) (Figure 8).

### Atanatolica decouxi sp. n.

Taxon classificationAnimaliaTrichopteraLeptoceridae

http://zoobank.org/BA4AF849-3B55-4347-B792-7EC60AF47C03

**Figure 5.**
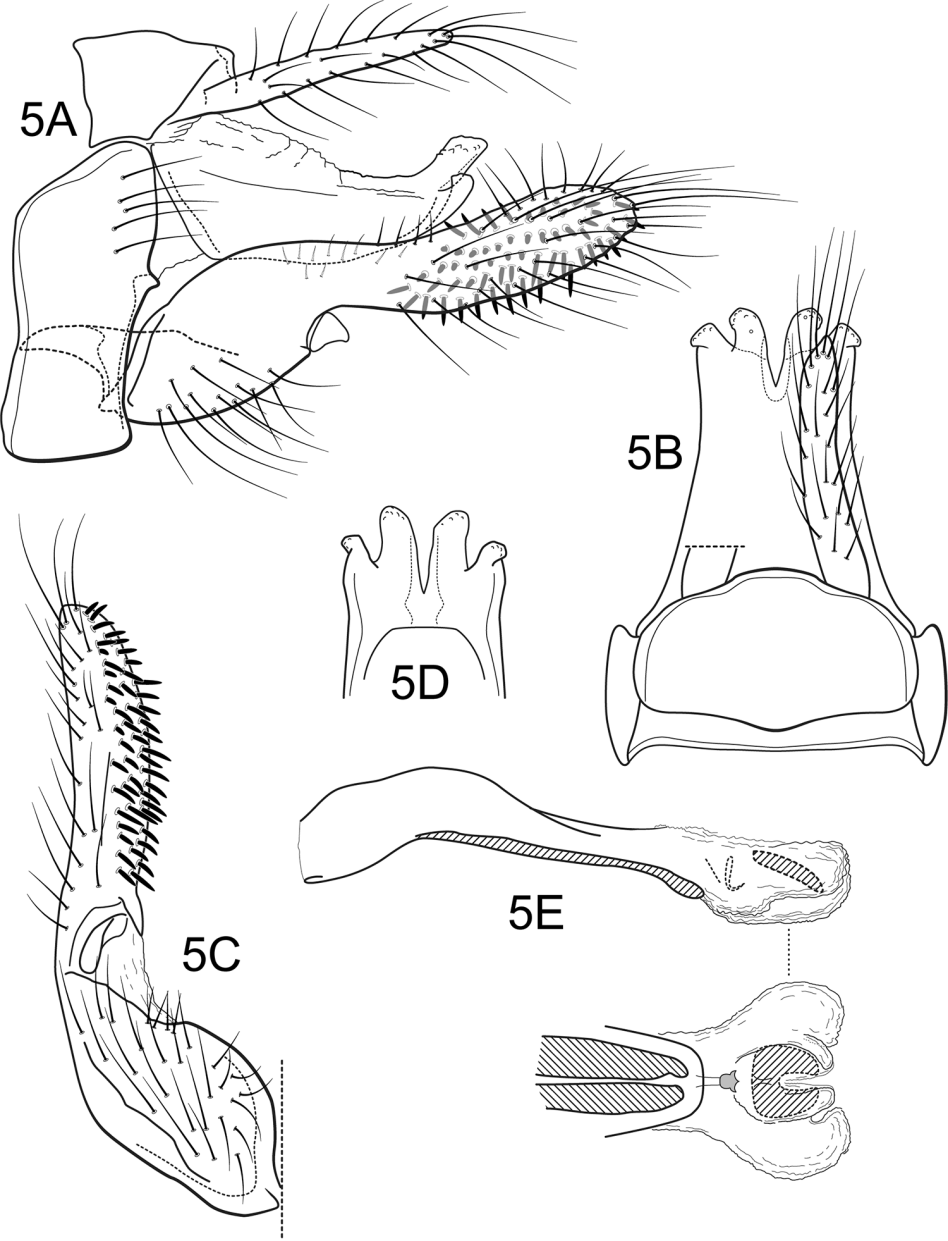
*Atanatolicadecouxi* sp. n., male genitalia. **A** Lateral **B** Segments IX and X, dorsal **C** Inferior appendage, ventral **D** Tergum X, caudoventral **E** Phallic apparatus, lateral (inset: phallic apparatus apex, ventral).

**Figure 6.**
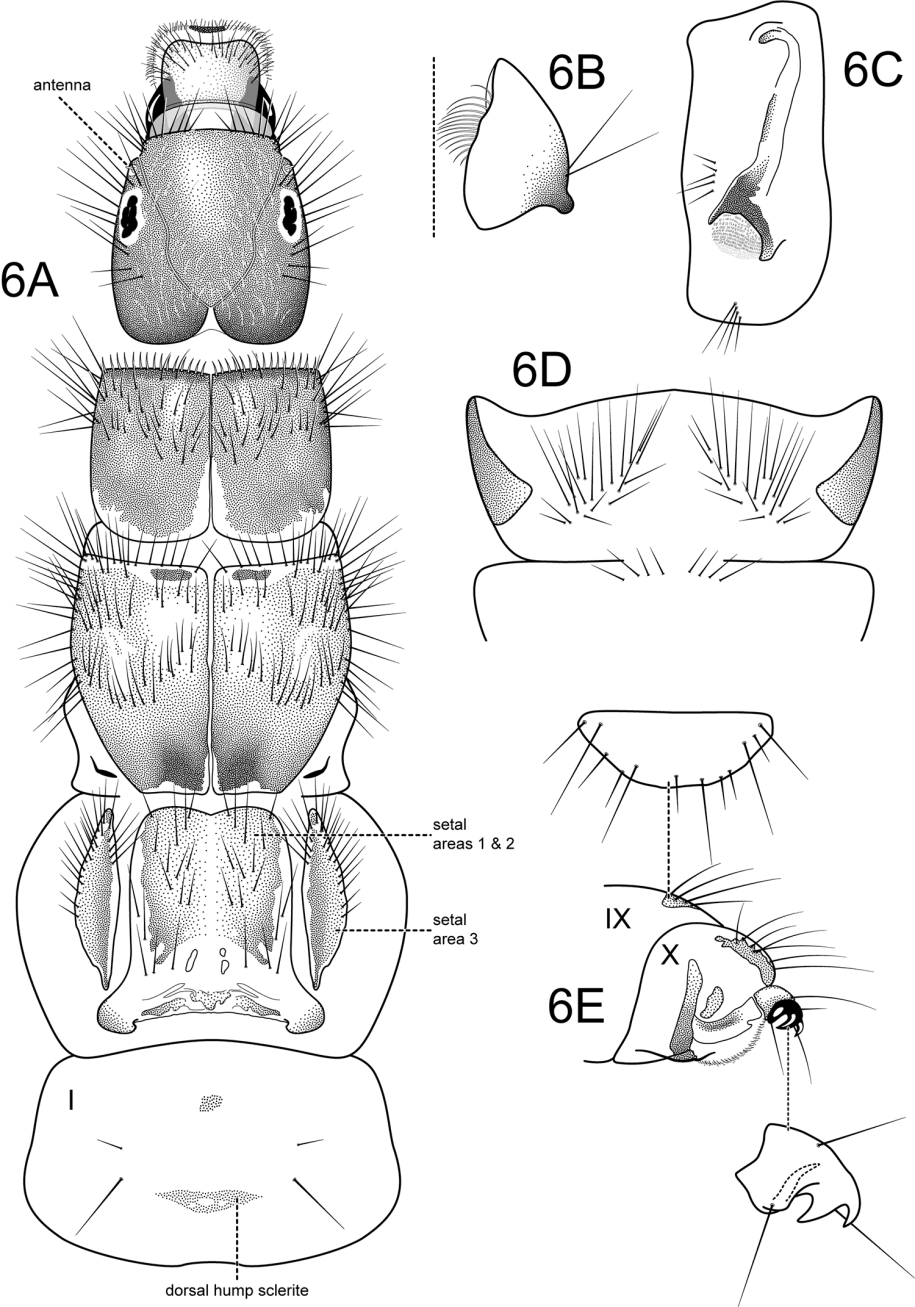
*Atanatolicadecouxi* sp. n., larva (tentative association). **A** Head, thorax, and abdominal; segment I, dorsal **B** Right mandible (enlarged), dorsal **C** Abdominal segment I, left lateral **D** Metasternum and abdominal segment I (partial), ventral **E** Abdominal segments IX and X, left lateral; upper inset: segment IX dorsal sclerite (enlarged), lower inset: anal claw (enlarged).

**Figure 7.**
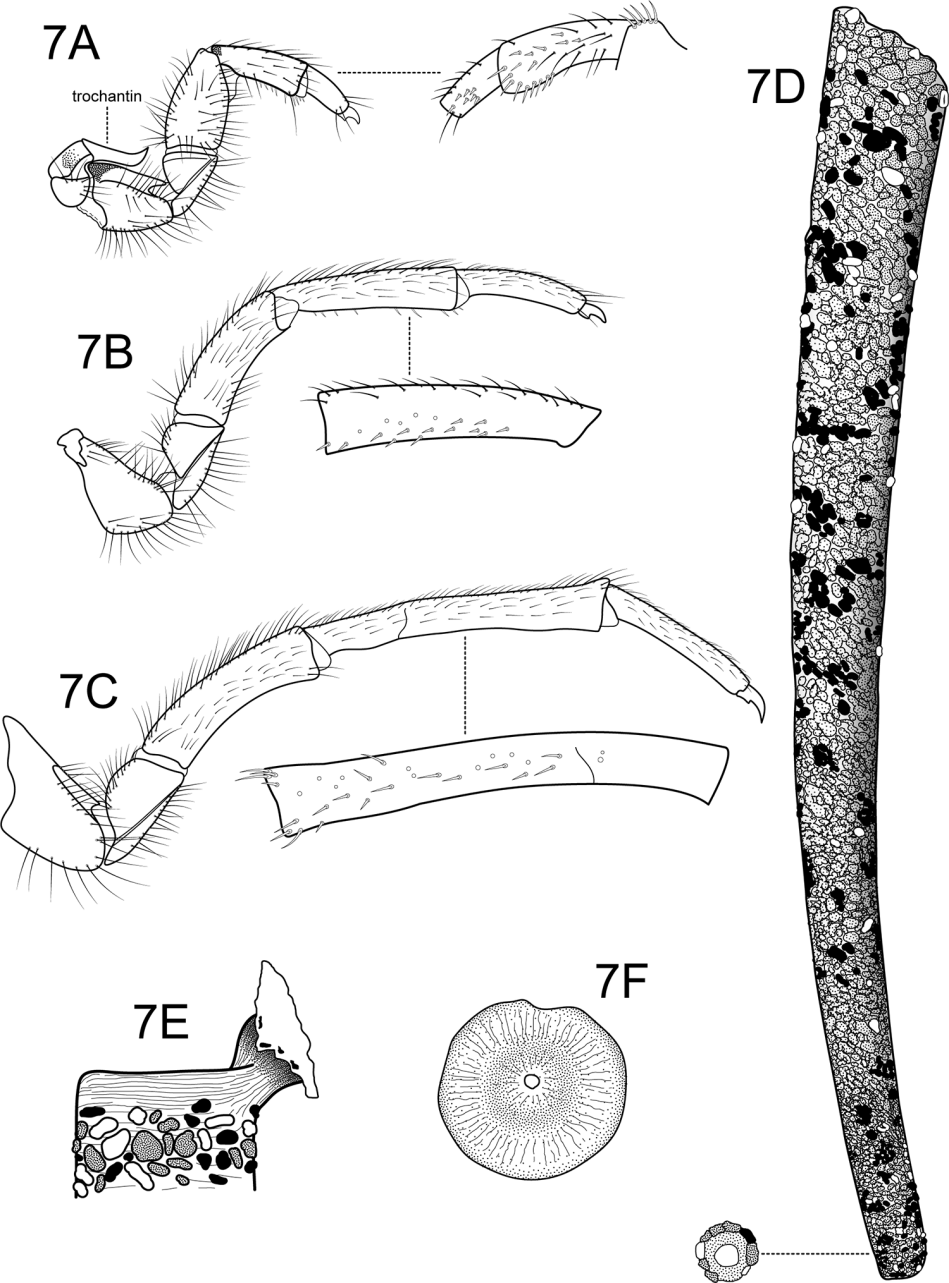
*Atanatolicadecouxi* sp. n., larva (tentative association). **A** Right foreleg (inset: fore tibia and tarsus, anterior surface) **B** Right midleg (inset: tibia, anterior surface) **C** Right hind leg (inset: tibia, anterior surface) **D** Larval case, lateral (inset: posterior opening) **E** Pupal peduncle **F** Pupal anterior silken cap.

**Figure 8.**
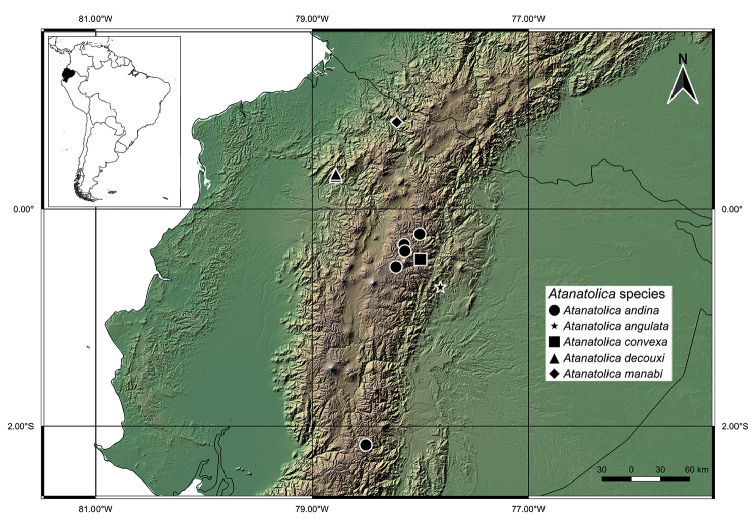
Distribution map of the new *Atanatolica* species described from Ecuador.

Figs 1B , 5 , 6 , 7 , 8

#### Diagnosis.

Morphology of the male genitalia of *A.decouxi* sp. n. is similar to *A.cotopaxi* Holzenthal, 1988 and *A.muyupampa* Holzenthal, 1988 from Ecuador and Bolivia, respectively. From *A.cotopaxi*, it differs by the slightly posteromesally produced segment IX, the longer preanal appendages reaching the apex of tergum X, and the shape and length of the apicolateral processes on tergum X, which are much shorter in *A.cotopaxi*. The putative larvae of *A.decouxi* sp. n. has spines on the anterior margin of the legs, similar to those found in *A.cotopaxi*, as illustrated by Holzenthal (1988), but the adults are light brown, whereas in *A.cotopaxi*, they are dark brown. *Atanatolicamuyupampa* differs from *A.decouxi* sp. n. by the shorter and laterally directed apicomesal processes on tergum X, and the deeper mesal cleft on tergum X between the apicomesal processes; in *A.decouxi* sp. n. both of these processes are apically rugose. Additionally, the inferior appendage in *A.decouxi* sp. n. is straight in ventral view, but curved mesad in *A.muyupampa*.

#### Description.

**Adult male.** Forewing length 11 ± 0.5 mm (n = 3). General color light brown, forewing membrane light brown, covered in brown setae along the costal margin and yellow setae through the remainder of the forewing. Head with yellow and light brown hairs. Antennae with light brown hairs on the scape and pedicel, flagellomeres with dark brown setae and ring of white setae basally. Maxillary palps light brown, with long, brown hairs. Thorax brown with yellow and brown hairs. Fore and midlegs with coxae and trochanter with light brown hairs, remaining segments with dark brown setae and ring of yellow hairs basally. Hind legs with yellow hairs and interspersed brown spines, increasing in thickness towards the tarsal segments. Tibial spur formula 0, 2, 2.

#### Genitalia.

Segment IX annular, short, with anterior margin sinuous, posterior margin slightly produced mesally (Figure 5A). Preanal appendages as long as tergum X, slender, digitate, setose (Figure 5A, B). Tergum X notched apicomesally, basal portion membranous, with two pairs of sclerotized apical processes; apicomesal processes digitate, slightly directed laterad in dorsal view (Figure 5B), apex rugose; apicolateral processes slightly shorter than apicomesal processes, thumb-like, directed laterad in dorsal and caudoventral views (Figure 5B, D), apex rugose. Inferior appendages with basal portion of first segment broad, setose, mesal margin rounded, apical portion digitate, almost straight in ventral view (Figure 5C), covered with stout, spine-like setae on its mesal surface; second article short, triangular, directed ventrad (Figure 5A, C). Phallic apparatus simple, without any processes; phallobase tubular; phallotremal sclerite complex, consisting of pair of elongated sclerites ventrally, hooked sclerite subapically, and U-shaped sclerite apically (subrectangular in lateral view); endothecal membranes trilobed (Figure 5E).

#### Larva.

Largest instars, assumed to be the 5^th^, up to 13.8 mm in length (n = 89).

Head (Figure 6A) ovate, brown; eyes large; antennae very short; coronal suture very short, broad; ventral apotome a single sclerite, elongate rectangular, unpigmented; head with long, prominent primary setae in postgenal region and along anterior edge of frontoclypeal apotome; parietal region and frontoclypeus covered with short, closely appressed, clear setae; labrum quadrate, prominent, with row of many short to long primary setae along anterior third and many very short secondary setae along membranous apical edge; mandibles broadly triangular, without separate teeth, with smooth mesal scraping edge, patch of curved setae in mesal concavity (Figure 6A, B). Thorax (Figure 6A): pronotum slightly longer than wide, brown, except for unpigmented posterior edge; covered with two sclerites, with many long setae dorsally and laterally on anterior half, anterior edge with row of uniformly spaced short, spine-like setae. Mesonotal sclerites almost completely covering mesonotum, brown, with pair of small elongate-oval darkly pigmented anteromesal marks; with many long setae dorsally and laterally on anterior half, mesal setae forming W-shaped row. Metanotal *sa*1 (*setal area* 1) and *sa*2 sclerites completely fused, forming large single median plate with posterolateral corners extended and directed medially, with brown pigmentation mesally and small patches of pigmentation along posterior edge; covered with long setae; *sa*3 sclerites long, oval, brown, except for narrow unpigmented mesal edge, with long marginal setae. Meso- and metapleural sclerites large, brown; metasternum (Figure 6D) with pair of ventrolateral patches of ca. 20 long setae. Foretrochantin horn-shaped (Figure 7A). Legs elongate, cylindrical, robust, brown, setose; foreleg the shortest, hind leg the longest; short, spine-like setae present on anterior (mesal) surface of tibia and tarsus of foreleg (Figure 7A and inset) and tarsi of midleg and hind leg, hind tibia with incomplete suture at basal third (Figure 7B, C, and insets); tarsal claws short, thick. Abdomen: long and slender, abdominal gills not apparent; segment I with small, elongate-oval dorsal sclerite and dorsolateral setae, one long, one short (Figure 6A); lateral hump sclerite (Figure 6C) of segment I prominent, elongate, ventral portion heavily sclerotized and encompassing membranous, raised area covered with minute setae, dorsal portion very lightly sclerotized and extending almost to dorsum of segment; with anteromesal and ventrolateral rows of ca. 4–5 setae; abdominal fringe sinuous, very narrow, composed of minute spicules; with small lateral tubercles on segment VIII (identical as those illustrated by Henriques-Oliveira and Santos 2012: fig 2H); dorsal sclerite of segment IX semicircular in dorsal view (Figure 6E upper inset), with ca. 12 alternating short and long setae along posterior edge; anal prolegs (Figure 6E) each with narrow ventral plate in addition to small lateral sclerite and ventral sole plate, dorsolaterally with darkly pigmented secondary lateral sclerite; band of uniform small spines adjacent to anal opening; anal claw with robust primary hook and single dorsal accessory hook (Figure 6E lower inset).

#### Larval case.

Elongate, narrow, gently curved and tapering, up to 20 mm long (Figure 7D); composed of small sand grains; posterior opening restricted to small opening by silken ring (Figure 7D inset); prior to pupation case fixed to substrate by short silken peduncle (Figure 7E), and anterior opening closed by silken cap with single opening (Figure 7F).

#### Remarks.

Larvae described here are tentatively assigned to *A.decouxi* sp. n. Unfortunately, no adult male metamorphotype pupae were collected to confirm the association. Larvae and adults were collected at the same site, but on different dates, and adults of only the single species were collected. In our previous collections of species in the genus and from museum material, it appears that species of *Atanotolica* do not co-occur at a site, lending support to this tentative association. The probable larva of *A.decouxi* is very similar to those described previously by Holzenthal (1988) and Henriques-Oliveira and Santos (2014). The larva of *A.decouxi* sp. n. described here is very similar to *A.nordestina* Henriques-Oliveira & Santos, 2014 in overall color and structure, but the morphology of the small spine-like setae on the anterior surfaces of the tibiae and tarsi may be distinctive; at least they are different from several species illustrated by Holzenthal (1988: figs 34–41) and most similar to those of *A.cotopaxi*. The lateral hump sclerite is also very similar to that described for *A.cotopaxi* by Holzenthal (1988: fig. 50) and *A.nordestina* (Henriques-Oliveira and Santos 2014: fig 2G). The case of *A.cotopaxi* is made of transparent silk with a few rock inclusions (Holzenthal 1988: fig 71), while those of *A.decouxi* sp. n. and *A.nordestina* are made entirely of rocks.

#### Holotype male.

**ECUADOR**: **Imbabura**: Reserva Los Cedros, Río de la Plata, 0.32495N, 78.7808W, 1587 m, 15.iii.2012, B Ríos-Touma, G Bragado, T Policha (UMSP) [UMSP000158717].

#### Paratypes.

**ECUADOR**: **Imbabura**: Reserva Los Cedros, Río de la Plata, 0.32495N, 78.7808W, 1587 m, 1♂, 1♀, 15.iii.2012, B Ríos-Touma, G Bragado, T Policha (UMSP), Reserva Los Cedros, tributary to Río Los Cedros, 0.30374N, 78.782W, 1312 m, 1♂, 2♀, 18-19.x.2011, R Holzenthal, B Ríos-Touma, A Encalada (MECN).

#### Additional material examined.

**ECUADOR**: **Imbabura**: Reserva Los Cedros, Río de la Plata, 0.32495N, 78.7808W, 1587 m, 84 larvae, 18.x.2011, R Holzenthal, B Ríos-Touma, A Encalada (MECN), 5 larvae (UMSP).

#### Etymology.

We dedicate this species to José DeCoux, an exceptional person who has been protecting Bosque Protector Los Cedros for more than three decades.

#### Distribution.

Imbabura Province (Ecuador) (Figure 8).

#### Natural history.

**Figure 9.**
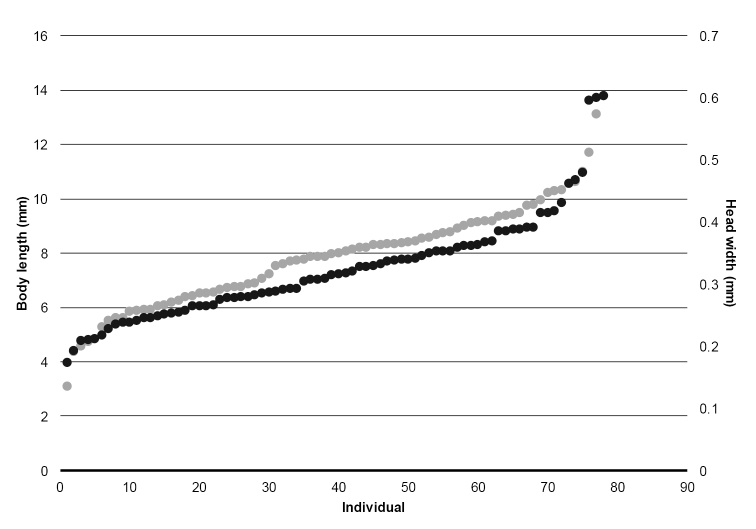
Body length (mm) (gray) and head width (mm) (black) of *A.decouxi* sp. n. larvae (n = 89) from Río de la Plata, Reserva Los Cedros, Imbabura Province, Ecuador.

Larvae were found in high densities in the Río de la Plata on rocks adjacent to a large pool and in the riffle below the pool, forming groups of individuals (Figure 1B). All larvae were submerged. Larvae were observed feeding by scrapping periphytic algae growing on rocks. We were not able to differentiate separate stages of larvae collected, but the size distribution suggests continuous growth in this tropical region (Figure 9).

##### New distribution record

***Atanatolicamanabi*** Holzenthal, 1988:83 [Type locality: Ecuador, Manabi, Santo Domingo de los Colorados (79 km W); NMNH; ♂].

**ECUADOR: Carchi**: Río Hualchancito near Hacienda Primavera, 0.80279N, 78.21816W, 1200 m, 1♂, 1♀, 11.ix.2017, B Ríos-Touma (UMSP) (Figure 8).

The species was previously recorded from three males and several series of larvae collected from “Santo Domingo de los Colorados” and vicinity by workers from the Smithsonian Institution in the mid-1970s. The specimens from Carchi represent the only additonal records of the species since those collections. The male genitalia are identical to those illustrated for the holotype by Holzenthal (1988: fig. 21). In the Carchi specimens, the wings of the strikingly colored adults are in perfect condition. The forewings are brown with a large patch of golden hairs on the apical half extending as a band along the posterior (anal) margin to near its base (dorsal on specimen when wings folded at rest). The apical edge of the forewing bears a fringe of bright, white hairs. The legs and antennae are banded with white and brown hairs.

## Discussion

The four species described here belong to the *Atanatolicadominicana* species group established by Holzenthal (1988) based on wing venation characters (fork I in the forewing distinctly petiolate). All but one of the species described here are known from only one locality, which suggests that members of the genus are diverse in the Andes. The only exception is *A.andina* sp. n., which is more widespread and occurs at the highest elevations recorded for the genus (3980 m). It likely occurs at other, high elevation sites along the Andes. This species, and the others described here, represent the first members of the genus described from Ecuador since Holzenthal’s (1988) study, indicating the lack of collecting and taxonomic research in a region undergoing environmental changes due to climate change, agriculture, and mining.

## Supplementary Material

XML Treatment for Atanatolica andina

XML Treatment for Atanatolica angulata

XML Treatment for Atanatolica curvata

XML Treatment for Atanatolica decouxi
